# Addressing the quality of paediatric primary care: health worker and caregiver perspectives from a process evaluation of PACK child, a health systems intervention in South Africa

**DOI:** 10.1186/s12887-021-02512-7

**Published:** 2021-01-28

**Authors:** Robyn Curran, Jamie Murdoch, Max Bachmann, Eric Bateman, Ruth Cornick, Sandra Picken, Makhosazana Lungile Simelane, Lara Fairall

**Affiliations:** 1 Knowledge Translation Unit, University of Cape Town Lung Institute, George Street, Observatory, Cape Town, Western Cape 7925 South Africa; 2 School of Health Sciences, University of East Anglia, Norwich, NR4 7TJ UK; 3 Norwich Medical School, University of East Anglia, Norwich, NR4 7TJ UK; 4 King’s Global Health Institute, King’s College London, London, SE1 9NH UK

**Keywords:** Paediatric primary care, PACK, Process evaluation, IMCI, Health systems strengthening, Educational outreach

## Abstract

**Background:**

The WHO’s Integrated Management of Childhood Illness (IMCI) has resulted in progress in addressing infant and child mortality. However, unmet needs of children continue to present a burden upon primary healthcare services. The capacity of services and quality of care offered require greater support to address these needs by extending and integrating curative and preventive care for the child with a long-term health condition and the child older than 5, not prioritised in IMCI. In response to these needs, the PACK Child intervention was developed and piloted in October 2017–February 2019 in the Western Cape Province of South Africa. We report health worker and caregiver perspectives of the existing paediatric primary care context as well as the extent to which PACK Child functions to address perceived problems within the current local healthcare system.

**Methods:**

This process evaluation involved 52 individual interviews with caregivers, 10 focus group discussions with health workers, 3 individual interviews with trainers, and 31 training observations. Interviews and focus groups explored participants’ experiences of paediatric primary care, perspectives of the PACK Child intervention, and tensions with implementation in each context. Inductive thematic analysis was used to analyse verbatim interview and discussion transcripts.

**Results:**

Perspectives of caregivers and health workers suggest an institutionalised focus of paediatric primary care to treating children’s symptoms as acute episodic conditions. Health workers’ reports imply that this focus is perpetuated by interactions between contextual features such as, IMCI policy, documentation-driven consultations, overcrowded clinics and verticalised care. Whilst these contextual conditions constrained health workers’ ability to translate skills developed within PACK Child training into practice, the intervention initiated expanded care of children 0–13 years and those with long-term health conditions, enhanced professional competence, improved teamwork and referrals, streamlined triaging, and facilitated probing for psychosocial risk.

**Conclusion:**

PACK Child appears to be catalysing paediatric primary care to address the broader needs of children, including long-term health conditions and the identification of psychosocial problems. However, to maximise this requires primary care to re-orientate from risk minimisation on the day of attendance towards a view of the child beyond the day of presentation at clinics.

**Supplementary Information:**

The online version contains supplementary material available at 10.1186/s12887-021-02512-7.

## Background

South Africa has not met the child mortality target for the Millennium Development Goals, in spite of having invested substantially in programmes and policies to achieve these targets [1]. In 2016, the child mortality and infant mortality rates were 42 and 35 per 1000, and their decline has slowed, making the target goals of < 25 and < 12 per 1000 live births, respectively, by 2030, a distant reality [2]. The World Health Organisation’s (WHO) Integrated Management of Childhood Illness (IMCI) strategy has played a crucial role in shaping primary healthcare for children under five in low and middle income countries (LMICs) for the past twenty years and has contributed to the decline in child mortality. Although it has seen many successes [3–7], implementation has been limited by inadequate local adaptation and infrequent revision of content, insufficient health worker training and supervision, and variable uptake in care [7]. In addition, IMCI focuses on priority life-limiting conditions like diarrhoea, pneumonia, HIV and TB, but does not address other common and increasingly pressing problems like asthma, allergies, epilepsy and mental illness. Lacking too is guidance for children over 5 years and management of long-term health conditions.

In the Western Cape province of South Africa almost every public sector primary care facility employs IMCI-trained nurses, who attend to the majority of children’s healthcare care needs. During consultation with clinical, managerial and policy stakeholders responsible for provincial paediatric health care, gaps in services for managing children at primary care level were identified, including the need to integrate routine care into the delivery of everyday care. This prompted the development of an expanded programme to address a larger remit of paediatric primary care. Led by the University of Cape Town’s Knowledge Translation Unit (KTU), the PACK Child intervention was developed, comprising a clinical decision support tool (the PACK Child guide), a cascade training and implementation programme, and health system strengthening components. This was based on the Practical Approach to Care Kit (PACK) Adult programme that has supported the delivery of comprehensive, integrated adult primary care in the province for the past 14 years [8, 9].

The PACK intervention aimed to get health workers to use the guide in their everyday practice and includes the PACK Child guide (localized for use in the Western Cape), health worker training and systems strengthening. The PACK Child guide collates and simplifies current evidence and policy for use in every nurse or doctor primary care contact with a child 0–13 years old [10]. Comprehensive in scope, it provides an approach to 63 symptoms and the routine care of 16 long-term health conditions, as well as a well child screen designed for every visit. The training programme is a streamlined version of PACK Adult training adapted from educational outreach [11] which entails nine onsite training sessions of 2 h highlighting alignment with IMCI, refresher training in growth monitoring, long-term health conditions, distribution of roles among health workers who see children and integration with documentation (e.g. Integrated Clinical Stationery, Road to Health Booklet- a caregiver held record of immunisations and child growth). Role clarification and documentation form part of the systems strengthening components, which also include a sensitisation session for facilities receiving referrals and clarification and compliance with IMCI prescriber levels.

Implementation of a health system strengthening intervention like PACK in a health system is a complex activity, requiring an understanding of how it will interact with the varying contexts of delivery. The Department of Health was especially concerned that we address stakeholder concerns of PACK’s integration with existing programmes and policies, particularly IMCI. To explore these issues and address concerns, a process evaluation was conducted alongside a pilot of PACK Child in 10 primary healthcare facilities in the Western Cape of South Africa to determine what refinements are needed at intervention and health system levels to optimise its implementation.

We have previously reported findings from observed consultations of how the implementation of PACK Child interacted with the wider context of paediatric care [12]. This paper complements those findings by reporting the perspectives of caregivers of children attending the facilities, and of health workers responsible for delivering the PACK Child intervention. These perspectives are lacking in literature on paediatric health care provision in primary health care settings in South Africa [13, 14].

## Methods

### Research setting

The setting for this pilot and process evaluation was 10 public- sector primary care facilities serving impoverished urban and rural communities in the Western Cape, South Africa*.* The Western Cape Health Department’s People Development Centre, which oversees training public sector healthcare workers in the Western Cape, purposively selected facilities for the study. They sought to provide maximum variation of primary care delivery, informed by whether clinics were Ideal Clinic sites, (a national policy to improve integration and quality of primary healthcare) [15]; had differing levels of PACK Adult training; and used recently developed checklist-enhanced child health records (Integrated Clinical Stationery). All facilities provided services both for the well child which includes growth monitoring and health promotion, immunisation, and care of the sick child. Different nurses conducted growth monitoring and health promotion (one enrolled nurse assistant (ENA)), immunisations (one enrolled nurse (EN) or professional nurse (PN)), or managed sick children (one or two IMCI trained PNs). One facility provided specialised clinics for asthma and skin conditions, and the other nine facilities reported rarely treating children with long-term health conditions.

**Fig. 1:**
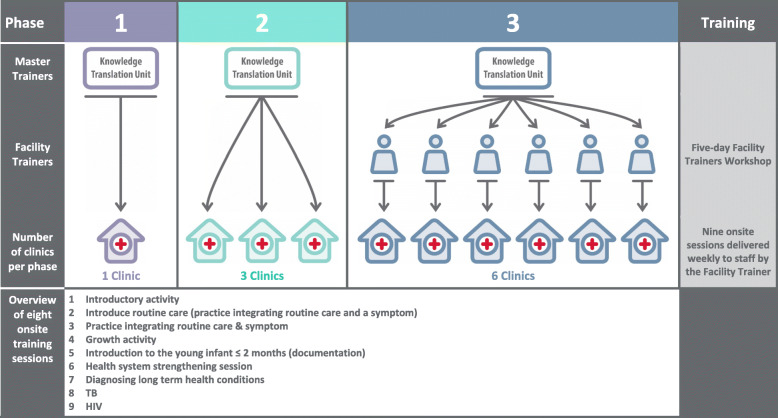
PACK Child training, implementation cascade model

The pilot was implemented in three phases **(**Fig. 1**);** Phase 1 was in one facility with training delivered by one KTU trainer (MS), Phase 2 in three facilities was implemented by two KTU trainers and Phase 3 in six facilities, where six facility trainers were trained to train staff within their facilities.

### Design and participant recruitment

The UK Medical Research Council guidance on process evaluation of complex interventions was used to inform the design, conduct and reporting of the study [16] as well as the Consolidated criteria for reporting qualitative research (COREQ) for the reporting of findings in this article [17]. The study used a mixed method approach including quantitative and qualitative data collection methods in all 10 primary care facilities. Quantitative data collection methods were training attendance logs and health workers questionnaires. Qualitative data collection methods were observations of training sessions, semi-structured interviews with caregivers, individual or focus group discussions with health workers and managers, and ethnographic observations of consultations and non-clinical areas.

In this paper we report findings from interviews with caregivers, focus group discussions with health workers, observations of training sessions and interviews with PACK Child trainers. To be eligible for inclusion, nurses and doctors needed to have received PACK Child training and caregivers and children to be receiving paediatric services at the selected facilities. Children needed to be aged 0–13 years to receive paediatric services. Purposive sampling was planned in Phase One to select and recruit a range of staff treating children, caregivers and their children. Sampling of children in Phase One was intended to be informed by diversity of conditions, level of deprivation and the age of the child. However, in practice, we recruited children presenting on that day, with clinic nurses identifying and approaching eligible participants in clinic waiting room areas. Findings from the analysis of Phase One qualitative observation (challenging aspects of using the PACK Child guide) and interview data (children’s presenting conditions), were used to inform theoretical sampling [18] of health workers, caregivers, children and timing of data collection in Phases Two and Three.

### Data collection

To understand caregiver perspectives of paediatric primary care and their experience of the PACK Child intervention, we conducted individual interviews with caregivers and their children at the facilities, either in the waiting area or in a consulting room where a room was available (Additional file 1). Where interviews were conducted in waiting areas, the interviewer identified sections of the waiting area that were less crowded and distant from other people. Caregivers were asked about their child’s health, their experience of paediatric primary care and changes in the care they received since the intervention. Caregiver interviews were carried out by RC or JM after their child’s consultation in which PACK Child was used. RC is a clinician and has experience working in primary care, as well as having extensive experience in conducting qualitative interviews in public health with vulnerable people. JM is a social scientist specialising in qualitative and mixed methods process evaluation. If the caregiver preferred to communicate in a language other than English, spoken by RC or JM, then a member of staff was asked to act as a translator. This only occurred on two occasions and the translator was a member of staff (receptionist) who was not involved in the clinical management of the patient, therefore an issue of bias was unlikely.

To understand the perspectives of users of the PACK Child guide, focus group discussions were conducted by RC with PACK Child-trained health workers at the completion of training at each facility. These were audio recorded. Health workers were asked about their perspectives of PACK Child training and implementation of the intervention in routine paediatric primary care, and its effect on clinic workflow and clinical competency (Additional file 2). All health workers’ focus groups were conducted in English. However, several health workers articulated their perspectives in Afrikaans, which we translated into English during transcription.

To understand features of the delivery of the PACK Child intervention in the varying contexts, a researcher (RC or JM) observed and took handwritten fieldnotes of all training sessions in Phase One, in order to record how training was delivered, how training was received, and points of difficulty within the training. To further investigate the interaction between intervention implementation and existing practice, we identified training sessions that evoked tensions between the PACK Child guidance and usual practice or raised challenges in attempts to integrate PACK Child guide into everyday routine care. Using these findings, we then selected other training sessions for observation in Phase Two and Phase Three. We conducted three interviews with trainers responsible for delivering the PACK Child training sessions, to elicit their perspectives of how health workers received the training and to pick up on points of difficulty identified in our earlier observations. These were important points of contrast to health worker perspectives of training sessions, elicited during health worker focus group discussions.

Data collection took place from October 2017 to February 2019. We conducted 52 caregiver interviews (Phase 1: 20; Phase 2: 12; Phase 3: 20), 10 health worker focus groups (one per clinic), 31 training observations (Phase 1: 8, Phase 2: 13; Phase 3: 10), and three trainer interviews. Interviews and focus groups had an average duration of 6 and 26 min respectively. The 10 focus groups had an average of six participants, were conducted in each of the facilities, and included doctors, Clinical Nurse Practitioners (CNPs), Professional Nurses (PNs), Enrolled Nurses (ENs) and pharmacists. All caregiver, health workers and trainer interviews were audio recorded and transcribed or translated in English.

The caregivers who were interviewed in the facilities had brought their children for a variety of problems including upper respiratory tract infections, skin problems, asthma, and eczema, or for immunisations. Caregivers in Phase One were interviewed throughout the nine-week period of the pilot. Analysis of these interviews revealed a lack of caregiver awareness of the PACK Child intervention, so we decided to conduct Phase Two and Three caregiver interviews towards the final session of the training programme, to allow more time for caregivers and children to be exposed to the use of the PACK Child guide in their clinic.

### Data analysis

Health worker focus group discussions, and caregiver and trainer interviews were transcribed verbatim and thematically analysed inductively [19], to understand how the PACK Child intervention was implemented from the perspectives of health workers and caregivers, and the interaction between the context of paediatric primary care and the intervention. We conducted open coding of the transcripts to reduce the data into fragments, which we then reflected upon through memos to begin to conceptualise properties and dimensions of categories and sub-categories which might form themes. We then carried out axial coding, examining how open codes related to each other in order to develop higher order categories. We also used a constant comparative method to test out categories, including searching for disconfirming cases [20]. Finally, we triangulated codes and categories with themes and field notes from our observations of training sessions, including comparing tensions and difficulties identified in observations with those reported by health workers and caregivers. This enabled us to identify key perspectives on current paediatric primary care and the extent to which PACK Child functions to address perceived problems within the current healthcare system. One researcher (RC) coded all of the interviews and focus group data in the first phase of the intervention, and a second (JM) independently coded 10% of the data. There was sufficient agreement between the coders with only minor disagreements in coding categories and choice of coding. These were discussed, with coding and coding categories refined as a result. Following completion of Phase 2 and 3, all coded data were reviewed by JM and RC to check for coding consistency. Minor inconsistencies were identified, discussed and recoded as appropriate.

This analysis enabled us to obtain a broad picture of the context of paediatric primary care according to the perspectives of health workers providing the care and the caregivers and children receiving it. It also highlighted how receipt of the PACK Child intervention interacted with these perspectives, enabling us to make specific recommendations for optimising implementation of the intervention more widely.

## Results

**Fig. 2:**
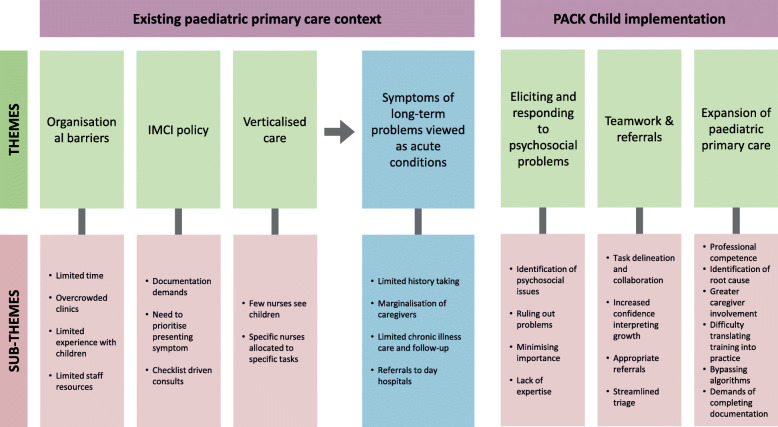
Themes and sub-themes

Our findings are separated into two broad categories. First, we report four themes from our analysis which provide insight into the paediatric primary care context prior to PACK Child implementation. These findings expand on our previous findings from consultation observations [12], that identified an institutionalised orientation to treat children’s symptoms as acute conditions, rather than as potential markers of underlying long-term health conditions. They provide insight into the wider context into which implementation of the PACK Child intervention was introduced. These four themes include: (i) organisational barriers, (ii) IMCI policy, (iii) verticalised care and (iv) symptoms of long-term problems viewed as acute conditions. Secondly, we present three themes from our analysis of the extent to which PACK Child addressed the perceived problems within the current paediatric primary care system. These are problems with: (i) expansion of paediatric primary care, (ii) teamwork and referrals and (iii) eliciting and responding to psychosocial problems. Figure 2 provides a visual representation of themes and sub-themes. The figure is not intended to represent the interaction between different contextual features and the PACK Child intervention, but to show contextual features that contributed to the institutionalised orientation to treat children’ symptoms as acute, episodic conditions, and how the introduction of PACK Child initiated a shift towards a different model of child care. To capture some of this complexity within the text here, we present the themes and sub-themes as part of a narrative rather than reporting each theme sequentially.

### Perceptions of paediatric primary care

Caregivers frequently shared experiences of care which indicated how health workers were oriented to treating children’s symptoms as acute episodic conditions. Despite having attended the facility repeatedly with the same problem, some caregivers reported that health workers rarely asked about the child’s previous history to help establish a diagnosis. Where caregivers did report a diagnosis of a long-term health condition, they provided accounts of an absence of ongoing management and routine follow up**.**

“The third son of mine, they say he got eczema, sometimes his skin would be so bad, and he would use all those creams. They would give you when his skin is got so bad, I would go to the clinic and they would give you the aqueous cream. Then they will say the skin is fine now. But when the child goes off medicine and it is finished, then I will stop going. Along the line the same thing will come back again. So, I was thinking maybe they are supposed to be giving me the same aqueous cream all the time, because you know he has eczema you know he should be coming for the same medication every time.”(Caregiver 50, Interview, Phase 1)

This finding from the perspective of caregivers was set against that of health workers who in many facilities reported that they rarely had children attending with long-term health conditions, and these children were attending larger clinics known in Cape Town as ‘day hospitals’.

“I haven't seen a child with long-term health conditions. I think most of them go to day hospital.” (Nurse, Focus Group 2, Phase 2)

A consequence of this orientation to acute, episodic care is that it limits the ability of health workers to address other problems that may have an important bearing on the child’s health more generally, as described by one caregiver with a child with behavioural problems.

“Earlier this year [Child’s name] schooling hasn't been going very well, we have tried to help him at home but it’s not easy as he struggles to concentrate and I thought maybe he has ADHD so I came to the clinic to ask for advice but nobody could really help me or give me proper information. They gave me this number and that number and this form and that form so that wasn't really proper information about how to have my child tested for ADHD, because if he does have it, I would like to do something about it before he gets older. But then I struggled up and down for a few months here and then I decided I am just going to leave it and I put more effort into helping him at home.”(Caregiver, Interview, Phase 3)

A recurrent sub-theme, often set alongside descriptions of health workers focusing on their child’s long-term health condition symptoms as acute problems, was the notion that caregivers felt marginalised as a resource and active agent in their child’s care, repeatedly describing how they were ignored, their view dismissed, or felt blamed for their child’s health problems.

“You know this nurse, sometimes they are very rude because they ask me "Why is your child like this, why is your child underweight” and it's not my fault and my child was sick and I was not giving him food. I was only breastfeeding. They always judge my child, why is your child like this, why is your child like this, why you don't feed your child. But they said to me I must not give the child water; I must not give the child food. You see. But they are rude sometimes, sometimes they are shouting you. If you don't have problem, you see. We come here to clinic because we want the help because the child is sick.” (Caregiver, Interview, Phase 1)

This sense of being ignored or blamed left caregivers feeling confused about their child’s condition and how to manage it. Yet, caregivers had a clear view of what they wanted to discuss:

“ … listen to a parent who come, as to what's been going on over the past couple of days, and why am I actually here, because I think that would be a good starting point, to say, ‘ok now do your routine check-up’. I find that to be a little bit of an issue sometimes, because I've been waiting for an hour.” (Caregiver, Interview, Phase 3)

In contrast to health workers, caregivers often conceptualised quality of care in terms of their level of participation in the consultation, indicating a need for caregivers’ voices to be heard and for their knowledge of their child to be a crucial part of determining the best care for their child that goes beyond dealing with the acute presentation on the day.

However, health workers reported numerous organisational barriers that limited their focus to acute symptoms, including having little time for routine care, limited health worker resources, limited experience in treating children, overcrowded clinics with long waiting times, requirements to prioritise critical questions about the presenting symptom as well as needing to conduct standard weight checks for calculating medication dosages.

“Sometimes it takes too long, because I work alone in the room, and I have about 30 patients, sometimes more the 30 that I see in a day, then it takes a while.” (Nurse, Focus group 10, Phase 3)

Further, health workers even questioned whether it was appropriate to offer more comprehensive care in this setting.

“When someone has been sitting here all day and the kid is screaming and they are sick, the mother is not psychologically in a space. I mean you can do a few, like look at the weight and do things that are red flags, that are critical, but you know the mother is not in a space, if she is sitting all day with a sick kid, to do a full comprehensive visit either.” (Doctor, Focus Group 8, Phase 3)

These problems were compounded by two broader contextual characteristics which shape how facilities deploy their health workers to care for children. First, verticalised care was the predominant pathway for children at all facilities with a limited number of nurses seeing children and each nurse was delegated to specific tasks.

“They have two dedicated nurses in child prep [triaging and weighing of children]. We've got someone dedicated for expanded immunization and PMTCT [prevention of mother to child transmission of HIV]. Then Mr ((name)) and Sr ((name)) render the child health.” (Manager, Interview, Phase 2)

Second was the role played by IMCI policy, which nurses felt played a fundamental role in structuring their consultations, underpinned by IMCI checklist documentation. The extent of this was illustrated by several nurses who reported that elements of the IMCI checklist were ingrained in their memory, including checking for danger signs, and ruling out cough, diarrhoea or ear pain.

“The IMCI, you know it by heart. You know it asks danger signs, it asks you cough, diarrhoea, ear.” (Nurse, Focus Group 2, Phase 2)

This method of consulting appeared to set fixed boundaries around the consultation, displaying a habituated practice that limits the possibility of including anything other than a discussion of acute symptoms, growth and feeding which the IMCI sequence follows.

“Because for the growth, you will only check when you are down here. Then you check what was my child’s weight. After you start with your symptoms...” (Nurse, Focus Group 1, Phase 1)

The use of standardised prompting limited the potential for caregivers to interject in the usual checklist process. This reflected our observations of consultations which displayed a predominance of questions designed to efficiently progress through a series of IMCI questions with limited caregiver involvement [12].

Taken together, the institutionalised focus of paediatric primary care to treat children’s long- term health condition symptoms as acute could be seen to be perpetuated by an interaction between IMCI policy, documentation-driven consultations with limited caregiver involvement and tracking of medical history, limited long-term health condition expertise and belief that children with long-term health conditions did not attend facilities, and a high demand for care with limited health workers resources. It was this organisational and social context into which the PACK Child intervention was introduced.

### The introduction of PACK child into paediatric primary care in the Western cape

#### Expansion of paediatric primary care

Despite reporting a number of organisational barriers which limited their focus to acute symptoms, health workers within all pilot facilities viewed the PACK Child’s training programme and guidance for conditions like HIV, tuberculosis, eczema and asthma as enabling their management and diagnosis of long-term health conditions.

“For example, I can say PACK guideline is very helpful, because I'm going to mention like skin symptoms. I used to see a child with a rash, but I couldn't differentiate what is it really, but when I go to the PACK Child, I know I can name it. It has got its specific diagnosis. I know what it is. But when I look at the PACK Child and I look at the child, then I see exactly what is in the PACK Child, and also what kind of treatment. It's very helpful.” (Clinical Nurse Practitioner, Focus Group 7, Phase 3)

This enablement of health workers practice was often linked to a sense of improved professional competence in being able to more effectively meet children’s needs, in this case in their ability to support children aged over 5 y.

“So, with PACK Child it's much better, you feel more secure that now you can treat the child until 12/13 years old.” (Clinical Nurse Practitioner, Focus Group 4, Phase 2)

A key component of the PACK Child intervention that health workers viewed as critical in supporting them to make a shift from acute symptom management to a view of the child’s treatment over time were the guide’s algorithms for guiding diagnosis, treatment and referral **(See** Additional file 3**for an example of the algorithms from PACK Child**). These views resonated with our observations of the PACK Child training sessions, where, through the medium of case scenarios, PACK Child trainers ‘scaffolded’ the skills of health workers by beginning with simple cases and then increasing the complexity in steps as they developed their competence in using the guide [21].

However, health workers expressed concerns about translating the skills developed within training sessions into their practice setting [12]. As the following extract from observational fieldnotes indicates, this difficulty led to a doctor reporting that she was hesitant about supporting nurses to prescribe inhaled asthma medication.

“In the asthma case presented in the session, the child presented with a recurrent wheeze for five days. The child was given a trial of an inhaler, but the clinicians omitted checking the bronchodilator response before prescribing. The doctor in the training felt that nurses would be prone to abuse inhalers if they were not assessing how previous episodes were managed and the correct diagnostic process followed including checking bronchodilator response.” (Field notes, observation of training session 7: Long-Term Health Conditions, Phase 3)

Here we see an interaction between the change that PACK Child is attempting to effect through guidance and training on how to treat long-term conditions, a primary care practice not habituated to check and track children’s medical history, an institutionalised focus to treat symptoms of long-term problems as acute conditions, and doctor’s perception that nurses would be ‘prone to abuse’ prescribing inhalers.

Despite this difficulty, the shift to enquiring about children’s medical history, considering root causes of their child’s condition, and tracking the course of long-term conditions, was appreciated by caregivers.

“What I say today is different because they never give us... you know when you are sick, they have to find the root of that, sometimes to go through what could be the cause of this, but they normally do a shortcut thing, especially here at the clinic. They just do the shortcut. So sometimes I see it keeps the baby, the baby keeps on suffering with the same thing because they never found the root of that. That's what I normally observe for myself.”(Caregiver, Interview, Phase 3)

This view of getting to the root cause was linked to extensive questioning that went along with using the PACK Child guide.

“Because it’s the third time. Sometimes you go to the doctor then the doctor says, just that one thing. Like this ((PACK Child guide)) was now nice. Everything was asked, and they have the patience to explain everything. And feel free to explain everything. Sometimes you go to the doctor you just cut you off because they rush you to get to another patient. Then that happens all the time.” (Caregiver, Interview, Phase 3)

Some caregivers described opportunities for them to explain their story and that health workers explained what was happening, indicating that they felt central to the decision-making process**.**

“Yeah, the way the doctor handled it. It was nice for me, because just for the fact that I can talk a lot of things ask lot of things. He come for his nose, but I could ask for this … she saw the marks of the eczema, almost like eczema.” (Caregiver, Interview, Phase 3)

However, the capacity for health workers to routinely provide this level of questioning was viewed as problematic by several health workers, despite caregivers noticing an increase in the depth of questioning and more opportunities for them to express themselves. Our observations of the PACK Child guide being used in consultations were that the perspectives of caregivers were rarely elicited [12]. Health workers explained that, because of the need to enquire more broadly in following PACK Child algorithms, as well as those of IMCI if the child was under 5 years, some questions and elements of algorithms were often bypassed. Instead health workers selected only what they considered was most appropriate or necessary in each consultation.

“Especially in clinics where we are strapped for time or short staff. Like today we have one sister doing all the sick children walking into the clinic. So, if she has to go through each little step, which is better, but she won't be able to see all the children. So, we tend to just skip to the problem, and ignore some of the routine.” (Doctor, Focus Group 10, Phase 3)

A frequently reported challenge for health workers in completing routine and symptom-based activities, as well as involving the caregiver and child in the consultation process, were the demands of completing Integrated Clinical Stationery alongside PACK Child.

“If you look at the paperwork, you write down on your IMCI form, you have to write down on your clinical as well, you have to write your script. We supposed to write in the child’s book ((Road to health book)), that we must be honest that is not done. You see how many different documents we need to write on. On top that, you starting to mix your medication, you are starting do all this. If you look at it, you more busy writing. You more focusing on writing instead of focusing on the child.” (Nurse, Focus Group 1, Phase 1)

#### Teamwork, patient flow and referrals

PACK Child was reported to have an impact on how health workers worked together and the referral of children. ENs, pharmacists, PNs and CNPs noted that the training helped to delineate different roles and responsibilities so that tasks were shared and enabled greater collaboration between health workers. This included a reconfiguration of which problems different cadres of health workers needed to manage, advise or oversee.

“What I have noticed is that she is always consulting, she is knowledgeable, almost to her utmost best of what is in PACK. But I have not seen her coming to me with a challenge she cannot go beyond. Even those kids that are referred to the emergency section. Sometimes, she doesn’t even come to me. She picks up the problem for the emergency and she send them without my intervention.” (CNP, speaking about an EN, Focus Group 2, Phase 2)

The “health system strengthening session”, which primarily focused on the flow of children through the clinic, facilitated changes in some facilities including one nurse deciding to weigh all children in consultation rooms rather than as a separate activity carried out by enrolled nurses. Some facilities reported that triaging of children was streamlined, and nurses felt equipped to identify children needing referral within the clinic.

“The other thing for triaging of the patients, the babies they really get emergency care much quicker, also their routine screening is so much easier with the length mat is there, everything is there. So, the staff have really benefitted from the training.”(Manager, Interview, Phase 2)

One of the training sessions was dedicated to embedding correct monitoring and interpreting growth in children. Nurses reported increased confidence in interpreting growth charts and alongside PACK Child screening tools they reported that they identified more children with problems that required referral. For this nurse, this was specifically in relation to identifying overweight children.

“We picked up lots of obese babies, of which now we are referring to the dietician and the dietician now has something to do. Before we were only picking up children with malnutrition. We didn't consider the obese, now we know when to refer, we know which weight is expected of each child, so we know when to refer. So now really it is of help.” (Nurse, Focus Group 2, Phase 2)

#### Eliciting and responding to psychosocial problems

According to health workers, use of PACK Child in consultations led to more psychosocial risk issues being identified in consultations, sometimes resulting in referral and resolution of these disclosures.

“It prompted you now with that section to ask for social problems. I also had one child: she didn't have an ID. Mum didn't have an ID that’s why she didn't register the child, and she can't apply for a grant, and I helped her. So that section is really good. It prompts you to ask those questions. In the past we overlooked it.” (Nurse, Focus Group 4, Phase 2)

However, our observations of consultations revealed that routine psychosocial risk questions, delivered in an embedded checklist approach to consultations were framed in a way to rule out problems instead of encouraging disclosure [12]. Furthermore, in some cases, where disclosures were made, the health worker could be seen to minimise its importance or not address the problem reported by caregivers. For one health worker the lack of expertise within facilities was a key reason why psychosocial issues are not fully addressed.

“We have a problem with psychiatrist. If you get the problem of abuse, then you must send the child to the hospital, because we don't have a person here every day, that's also a problem. Sometimes when you book the people, for that then the guy cancels his visit.” (Nurse, Focus Group 9, Phase 3)

## Discussion

The PACK Child intervention was developed in part to expand the scope of practice provided by IMCI, by including provision of paediatric primary care from under-fives to children aged up to 13 years and those living with long-term health conditions [10]. However, this study illustrates the challenges of implementing PACK Child to change an existing primary healthcare system that focuses on acute symptoms in a verticalised pathway of care with severe time limitations. This challenge is compounded by consultations that are often driven by complex and multiple demands for documentation completion, reducing meaningful interaction with the caregiver and child. These challenges need to be addressed in seeking to provide care for acute and long-term health conditions of children that is comprehensive and person-centred rather than nurse and documentation centric.

Caregivers in the study emphasised that the focus of paediatric primary care is on the primary presenting symptom, with little reference to past medical history resulting in repeated visits for the same presenting symptom. These perspectives were corroborated with findings of observations of consultations in this study [12] and with perspectives of health workers who reported an acute symptom focus of care and a preference for following IMCI from memory or according to a checklist. These findings also resonate with similar findings from a study of health care worker adherence to IMCI in Tanzania where nurses delivered the IMCI protocol from memory [22] and a study evaluating health care worker adherence to IMCI guidelines in South Africa, which identified that less than 2% of health care workers referred to IMCI guidelines during a paediatric visit [14].

Caregivers’ perceptions of paediatric primary care as non-participatory and positioning them as passive recipients of care was compatible with how health workers viewed consultations as dominated by completing required documentation. These findings are in keeping with a study in South Africa, which evaluated the change in quality of care provided to sick children as a result of routine implementation of IMCI which showed limited caregiver knowledge regarding medication or when to return to the facility [13].

These insights inevitably reveal the current state of paediatric primary care in the facilities included in this study, which could be generalisable to the broader continent where IMCI is also institutionalised within primary healthcare facilities and frame the way in which children are treated. This has unknowingly impacted on which questions are prioritised in a consultation and evidence from this study shows that the demands of completing the correct documentation drives this process rather than the caregiver and the child.

Our findings indicate that PACK Child has improved clinical knowledge and practice in the diagnosis and management of children, identification of long-term health conditions, and management of children above five years of age where previously guidance was limited. In some facilities it also catalysed more streamlined triaging and appropriate referrals, indicating the potential of PACK Child for enhancing the ability of clinicians to treat a wider range of conditions within facilities whilst also reducing the burden on emergency care services. However, in order for PACK Child implementation to be optimised within facilities, paediatric primary care needs significant restructuring to support its implementation at scale. There is a need for district and sub-district departments of health to prepare the health system through managerial buy-in, to support a different view of caring for children over time, changing prescribing regulations for professional nurses and to re-assess documentation [12], patient flow and referral pathways. This could be supported by refining the structure of the PACK Child guide to further improve use and facilitate a smoother consultation flow, to increase the focus on ongoing care of the child and identification of psychosocial issues. Rephrasing of parts of the PACK Child guide could facilitate greater involvement of caregivers within consultations.

### Strength and limitations

This process evaluation has included the perspectives of caregivers as well as health workers, which are rarely reported, providing critical insights on the current state of paediatric primary care in low income settings of the Western Cape, South Africa. However, partly as a consequence of the interview schedule, caregivers and health workers tended to separate out their perspectives of the wider paediatric context from their views of PACK Child which we then had to reintegrate through our analysis. However, the breadth of data we obtained from observations of training sessions, interviews and focus groups, which also follow on from our analysis of consultation data [12], provided opportunities for triangulating and extending our interpretations of the relationship between different contextual features and delivery of PACK Child. The perspectives of caregivers and health workers therefore add an important contribution for understanding the potential of PACK Child to fill important gaps in service provision. Such understanding of the interaction between the wider primary care context and implementation of PACK Child, generated from working inductively with different data, resonates with broader theoretical models of behaviour, such as Bronfenbenner’s socio-ecological model, as well as Implementation Science frameworks (e.g. Consolidated Framework for Implementation Research) [23], thereby providing a platform for future research to investigate how best to optimise and scale-up implementation across a diversity of primary care settings.

Caregiver interviews were often conducted in facility waiting rooms either before or following the child’s consultation, which limited the ability to have extended private discussions with caregivers. Although we took steps to ensure interviews with caregivers were conducted in spaces where they couldn’t be overheard, it is possible that this affected what caregivers reported. Children were often present during the interviews, 19 were aged over 5 years and their presence may have influenced what caregivers discussed during the interviews. Sampling of caregivers and children was also limited by those who attended on the day, which may have restricted the broader view of other caregivers with different problems who may have attended on different days and attended the clinic regularly. Our findings are also limited by needing to collect data both during and immediately following completion of the PACK Child training programme, which allowed little time for the intervention to be embedded into everyday practice. We tried to address this in Phase 2 and 3 by conducting interviews at the end of the training programme. The generalisability of this study was that it was conducted in the Western Cape Province of South Africa, which is arguably better resourced than other provinces in the country. Despite this, IMCI policy is pervasive across South Africa and other LMICs with policy targets to reduce mortality in under 5 s, underpinned by provincial documentation that attempts to standardise care of these children. The identification of an institutionalised orientation to view symptoms of long-term problems as acute conditions is likely transferrable to other settings and maybe exacerbated where the tensions between limited skilled resource and demand for care are more acute.

## Conclusion

The Sustainable Development Goal aim to significantly reduce child and infant mortality by 2030 using IMCI policy has shown some promise, however without significant changes at a health systems level this target may be unachievable. PACK Child offers support for this process by aiming to improve clinical skills for managing the broader needs of children, including long-term health conditions, strengthening teamwork and appropriate referral, and the identification of psychosocial problems. However, maximising the potential of PACK Child requires paediatric primary care to re-orientate from an acute episodic approach of the child, to the broader picture of the child’s health over time.

## Supplementary Information

**Additional file 1.** Semi-structured Interview Guide with Caregivers-data collection instrument for interviews with caregivers.

**Additional file 2.** Semi-Structured Focus Group Discussion Guide with Health workers-data collection instrument for focus groups with health workers

**Additional file 3.** PACK Child Algorithm-example of PACK Child symptom page.

## Data Availability

This is a process evaluation mirroring the context of paediatric primary care in the Western Cape, South Africa. Making the full data set publicly available could potentially be a breach to the privacy that participants were promised upon request of participation. In addition, our ethics approval from the Western Cape Department of Health, the City of Cape Town and University of Cape Town’s Human Research Ethics Committee was granted based on the anonymity of the individual consenting to participate. Due to these conditions, the authors are unable to make the full transcripts available to a wider audience. Excerpts of specific segments of the text will be reviewed for any potential identifying details and made available to researchers or reviewers who complete a data sharing agreement and abide by strict confidentiality protocols. Data requests may be sent to the corresponding author**.**

## Abbreviations

CNP: Clinical Nurse Practitioner
EN: Enrolled Nurse
ENA: Enrolled Nursing Assistant
FGD: Focus Group Discussion
HIV: Human Immuno-deficiency Virus
IMCI: Integrated Management of Childhood Illness
KTU: Knowledge Translation Unit
LIC: Lower -income country
PACK: Practical Approach to Care Kit
PN: Professional Nurse
RtHB: Road to Health Booklet
TB: Tuberculosis
WHO: World Health Organisation
