# Supplementary material for: Addressing the quality of paediatric primary care: health worker and caregiver perspectives from a process evaluation of PACK child, a health systems intervention in South Africa
Source: BMC Pediatr. 2021 Jan 28;21:58. doi: 10.1186/s12887-021-02512-7 (PMC7842050; doi:10.1186/s12887-021-02512-7)
Supplement: Supplementary file 1 — Additional file 1. Semi-structured Interview Guide with Caregivers-data collection instrument for interviews with caregivers. [file 12887_2021_2512_MOESM1_ESM.pdf]

PACK CHILD Study
Caregiver/Child Interview Guide

Introduction:

- Thank the patients for agreeing to meet/their time etc.
- Outline the study aims and aims of this discussion is to:
  - (i) Understand the caregivers living situation and what life is like and how this affects their child's health.
  - (ii) Tell them that you have some questions but really the person can say whatever or however much they like, whatever they feel is important to say.
  - (iii) Check patients have read/understood the study information leaflet. Remind them about confidentiality and anonymity.
  - (iv) Also explain your role as interviewer, you don't work for the hospital where the parent/caregiver/child is being treated and have no influence on their care.
  - (v) Reiterate that this is about understanding the parent/caregiver/child experience, whatever that may be and that what they say will be really important for informing how others are treated in the future.
  - (vi) Advise interviewees that we seek their experience/views, with no 'right' or 'wrong' answers.
- Give them a chance to ask questions.
- Check that they wish to proceed with the interview.
- Complete/sign two consent forms (leave one with caregiver/parent).
- Advise them they can stop the interview at any point/decline to answer any questions.
- Advise them when audio-taping will begin.

Interview questions:

I. GENERAL LIVING SITUATION AND FAMILY

Can you tell me about your living situation and family and how this may impact on your child's health?

II. CHILD HEALTH HISTORY

Can you tell me about your child's health and what health conditions they live with?

Prompts

- history of each condition; how long had, when and circumstances of diagnosis. Aim to get a narrative or a biography.
- current living circumstances and how this has changed in the history of their condition(s)

III. PAST HEALTH SERVICE EXPERIENCE

How has your child been treated up till now

Prompts

- Who did the parent/child seek help from initially? What happened?
- How did they obtain access to help?
- What treatments were they given? What was that like?
- What has changed since then?

IV. CURRENT EXPERIENCE

If they have had a consultation either with or without PACK Child:

Prompts

- Who have you seen recently to get help? Why did you see them? What happened?
- Do you remember what questions they asked? Were you able to answer the questions?
- What advice/treatments were you given?
- Have you got another appointment arranged here or anywhere else?

Possible prompts/ follow-up questions for all topics:

- Are you able to tell me more about that?
- Can you give an example of a time when that happened/ when you felt like that?
- How did that make you feel?

- What did you think about that?
- Just talk about whatever comes to mind
- What happened (next)?
- Do you mean …?

Would you like a summary of what comes out of the conversations?
Thank you for your time.

These topic guides provide a structure for the interviews. As with most qualitative research studies, it may be necessary for interviewers to modify questions slightly depending on the individuals being interviewed, and to ask additional probing questions directly relevant to the main topics covered in the topic guide. Questions may not follow the exact order outlined in the topic guides if interviewees raise issues without prompting'.
