# Supplementary material for: Addressing the quality of paediatric primary care: health worker and caregiver perspectives from a process evaluation of PACK child, a health systems intervention in South Africa
Source: BMC Pediatr. 2021 Jan 28;21:58. doi: 10.1186/s12887-021-02512-7 (PMC7842050; doi:10.1186/s12887-021-02512-7)
Supplement: Supplementary file 2 — Additional file 2. Semi-Structured Focus Group Discussion Guide with Health workers-data collection instrument for focus groups with health workers [file 12887_2021_2512_MOESM2_ESM.pdf]

Staff Interview Guide: PACK Child Process Evaluation

Introduction:

- Thank staff member for agreeing to meet/their time etc.
- Outline the study aims and aims of the interview. Reiterate confidentiality
- Check staff have read/understood the study information sheet.
- Give them a chance to ask questions.
- Check that they wish to proceed with the interview.
- Complete/sign two consent forms (leave one with staff).
- Advise staff that we seek their experience/views and importance of getting insight to improve delivery of the intervention beyond the study.
- Advise them they can stop the interview at any point/decline to answer any questions.
- Advise them when audio-taping will begin.

Interview questions:

I. Background/Experience

Can you start by telling me a bit about your professional background and how you came to be involved with the PACK Child study?

Prompts

- Do you work with children as part of your normal job role? If so in what capacity?
- Have you received any specific training in child health?

II. Study Role

How would you describe your role in this study?

Prompts

- How has the study affected the other tasks in your role at the clinic? What problems did you face? How did you manage any difficulties?
- How has this Study fitted in with your usual working pattern? Additional hours? Payment for overtime?

III. Thinking about the set-up phase of the study

How well do you feel the training prepared you for the study?

Prompts

- Who did your training- KTU trainer, master trainer, facility trainer?
- Were you able to attend all the training sessions?
- What feedback would you give on the guideline training?

Can you tell me about your experience of communicating with the study team? How was that?

IV. Guideline Use

Can you describe your experience of using the PACK Child guideline.

Prompts

- How have you found it useful in your consultations?
- What facilitates your ability to use the PACK Child guideline during a consultation?
- What are the barriers to using the PACK Child guideline?

V. Organisational

Have there been there any organisational problems that have influenced your use of PACK Child?

Prompts

- Have there been any resource issues? E.g. lack of guidelines, understaffed?

VI. Sustainability

Do you think that the PACK Child Guideline will continue to be used once the study is over?

Prompts

- How feasible do you think it would be for staff to use the PACK Child guideline in their consultations daily?
- What would prevent staff from using the guideline?
- What could encourage staff to use the guideline?

When you started the study, what did you expect might happen? To what extent were your expectations met?

Is there anything else you'd like to say about your experience of participating in the study?

- prompt – experience; suggestions; study requirements;
overall 'feel' of the process etc

Prompts/ follow-up questions:

- Are you able to tell me more about that?
- Can you give an example of a time when that happened/ when you felt like that?
- How did that make you feel?
- What did you think about that?
- Just talk about whatever comes to mind
- What happened (next)?
- Do you mean …?

Would you like to see a summary of what comes out of the interviews with staff?
Thank you for your time.
