# Supplementary material for: Addressing the quality of paediatric primary care: health worker and caregiver perspectives from a process evaluation of PACK child, a health systems intervention in South Africa
Source: BMC Pediatr. 2021 Jan 28;21:58. doi: 10.1186/s12887-021-02512-7 (PMC7842050; doi:10.1186/s12887-021-02512-7)
Supplement: Supplementary file 3 — Additional file 3. PACK Child Algorithm-example of PACK Child symptom page. [file 12887_2021_2512_MOESM3_ESM.pdf]

Pilot version - not for distribution

Cough and/or breathing problems

The child with breathing problems may have noisy breathing, wheeze, grunting, snoring or stridor (noisy, high-pitched breathing). If child not breathing ↩24.

Give urgent attention to the child with:

- Baby < 2 months old
- History of apnoea (episodes of no breathing > 10 seconds)
- Unable to drink/feed
- Tires/sweats during feeds
- Lower chest indrawing
- Nasal flaring
- Grunting
- Stridor
- Blue lips/tongue
- Sats ≤ 92%
- Restless or irritable
- Lethargy or decreased level of consciousness (↩135)
- Sudden difficulty breathing and any of: generalised itchy rash, face/tongue swelling, fainting/dizziness/collapse, abdominal pain/vomiting or exposure to likely allergen[1], check for **anaphylaxis** ↩112.

Manage and refer urgently:

- Give oxygen 2L/minute via nasal prongs. If < 1 year old with blocked nose, instil sodium chloride 0.9% solution 1 drop into each nostril and suction the nose.
- If wheeze →51 or if known heart problem →117.
- Check fingerprick glucose: if ≤ 3 or ≥ 11mmol/L ↩31.
- Give ceftriaxone 80mg/kg (up to 1.5g) IV/IM (↩129:7). If < 1 year old and HIV positive or unknown, also give single dose of co-trimoxazole (↩130:13).
- If stridor, encourage carer to keep child calm.
  - Give prednisone 2mg/kg (up to 40mg). Give epinephrine[2] (1:1000) 1mL in 1mL sodium chloride 0.9% via nebuliser (oxygen 8L/minutes) every 15 minutes until stridor disappears.

Approach to the child with cough and/or breathing problems not needing urgent attention:

- If smoking in the house, alert to risks and encourage smoker to quit ↩PACK Adult helpline.
- If recent episode of choking, **inhaled foreign body** likely. Refer same day.
- If current wheeze →51.
- If breathless on exertion, discuss/refer same day.
- If coughing attacks with "whoop" on breathing in, **pertussis** likely: give azithromycin 10mg/kg (up to 500mg) once daily for 5 days (↩129:6), notify and isolate for 2 days.
- Ask about duration and number of episodes:

1 episode of cough and/or breathing problems lasting < 2 weeks
Is respiratory rate increased (↩135)?

Yes:

Pneumonia likely
- Give amoxicillin[3] 45mg/kg/dose (up to 1g) 12 hourly for 5 days (↩128:3).
- If > 2 episodes/year needing hospital, do HIV test if status unknown, and refer/discuss non-urgently.
- Review after 2 days: if respiratory rate still increased (↩135), refer.

No:

Runny/blocked nose

Common cold likely
- Check ears ↩45, throat ↩47, nose ↩46.
- Reassure carer antibiotics not needed.
- Advise to drink warm liquids to relieve symptoms.

Barking cough, may be hoarse

Viral croup likely
- Give single dose prednisone 2mg/kg.
- Advise to return immediately if worse or stridor develops.

Cough and/or breathing problems ≥ 2 weeks or repeated episodes
- If HIV status unknown, test for HIV ↩105.
- Exclude TB ↩100. While excluding TB consider other causes:

If recent common cold
- If wet cough ≥ 4 weeks, refer.
- If dry cough, **post-infectious cough** likely: should resolve by 8 weeks.

If blocked nose or noisy breathing worse at night and/or snoring ↩46.

If known with long term health condition:
- Asthma ↩114.
- Bronchiectasis ↩116.
- Heart problem ↩117.
- If life-limiting illness, also give palliative care ↩123.

If repeated episodes of cough, wheeze, tight chest or difficulty breathing ↩53.

Refer if cause uncertain or not growing well, chest deformity, cough > 8 weeks, coughs/chokes with feeds or cough worse despite treatment.

[1]Allergen can be an insect bite, ingesting medicine or new food in the last hour. [2]Epinephrine is also known as adrenaline. [3]If penicillin allergy (history of anaphylaxis, urticaria or angioedema), give azithromycin 10mg/kg (up to 500mg) once daily for 3 days instead (↩129:6).
